# Gene duplication, rather than epigenetic changes, drives FGF4 overexpression in KIT/PDGFRA/SDH/RAS-P WT GIST

**DOI:** 10.1038/s41598-020-76519-y

**Published:** 2020-11-16

**Authors:** Milena Urbini, Annalisa Astolfi, Valentina Indio, Margherita Nannini, Angela Schipani, Maria Giulia Bacalini, Sabrina Angelini, Gloria Ravegnini, Giovanni Calice, Massimo Del Gaudio, Paola Secchiero, Paola Ulivi, Elisa Gruppioni, Maria Abbondanza Pantaleo

**Affiliations:** 1 Biosciences Laboratory, Istituto Scientifico Romagnolo per lo Studio e la Cura dei Tumori (IRST), IRCCS, Meldola, Italy; 2 Department of Morphology, Surgery and Experimental Medicine, University of Ferrara, Ferrara, Italy; 3 “Giorgio Prodi” Cancer Research Center (CIRC), University of Bologna, Bologna, Italy; 4 Division of Oncology, Azienda Ospedaliero Universitaria di Bologna, Via Albertoni 15, Bologna, Italy; 5 Department of Experimental, Diagnostic and Specialized Medicine, University of Bologna, Bologna, Italy; 6 IRCCS Istituto delle Scienze Neurologiche di Bologna, 40139 Bologna, Italy; 7 Department of Pharmacy and Biotechnology, University of Bologna, Bologna, Italy; 8 Laboratory of Preclinical and Translational Research, IRCCS-Referral Cancer Center of Basilicata (CROB), 85028 Rionero in Vulture, PZ Italy; 9 Department of Organ Insufficiencies and Transplantation, General Surgery and Transplantation, S. Orsola-Malpighi University Hospital, 40138 Bologna, Italy; 10 Laboratory of Oncologic Molecular Pathology, S. Orsola-Malpighi Hospital, Bologna, Italy

**Keywords:** Cancer genomics, Transcription, DNA methylation, Gastrointestinal cancer

## Abstract

Gastrointestinal stromal tumours that are wild type for KIT and PDGFRA are referred to as WT GISTs. Of these tumours, SDH-deficient (characterized by the loss of SDHB) and quadruple WT GIST (KIT/PDGFRA/SDH/RAS-*P* WT) subgroups were reported to display a marked overexpression of *FGF4*, identifying a putative common therapeutic target for the first time. In SDH-deficient GISTs, methylation of an FGF insulator region was found to be responsible for the induction of FGF4 expression. In quadruple WT, recurrent focal duplication of *FGF3*/*FGF4* was reported; however, how it induced *FGF4* expression was not investigated. To assess whether overexpression of *FGF4* in quadruple WT could be driven by similar epigenetic mechanisms as in SDH-deficient GISTs, we performed global and locus-specific (on *FGF4* and FGF insulator) methylation analyses. However, no epigenetic alterations were detected. Conversely, we demonstrated that in quadruple WT GISTs, *FGF4* expression and the structure of the duplication were intimately connected, with the copy of *FGF4* closer to the *ANO1* super-enhancer being preferentially expressed. In conclusion, we demonstrated that in quadruple WT GISTs, *FGF4* overexpression is not due to an epigenetic mechanism but rather to the specific genomic structure of the duplication. Even if *FGF4* overexpression is driven by different molecular mechanisms, these findings support an increasing biologic relevance of the FGFR pathway in WT GISTs, both in SDH-deficient and quadruple WT GISTs, suggesting that it may be a common therapeutic target.

## Introduction

Approximately 10–15% of gastrointestinal stromal tumours (GISTs) do not harbour mutations in *KIT* or *PDGFRA* and are referred to as KIT/PDGFRA wild-type (WT)^1^. In general, WT GISTs are more resistant to imatinib, and a proper therapeutic strategy is still lacking. Of these cases, 20–40% are characterized by the loss of SDHB (SDH*-*deficient GIST)^2–4^, approximately 15% carry *BRAF/RAS* or *NF1* mutations, and the remaining cases are designated as KIT/PDGFRA/SDH/RAS-P WT GISTs (or quadruple WT GISTs) in which the molecular driver alterations are still unknown.

In recent years, many efforts have been made to characterize quadruple WT GISTs; however, whole-exome sequencing uncovered considerable molecular heterogeneity in this subgroup of patients despite a homogeneous gene expression profile^5^. Nonetheless, alterations in *FGFR1* (point mutations and fusion genes) and *FGF4* (duplication and overexpression) have been detected in several cases, supporting the idea that the FGFR pathway could play a relevant role in the biology of quadruple WT GISTs^5–7^. Interestingly, overexpression of *FGF4* and activation of its signalling were recently reported in SDH-deficient GISTs^8^. In these cases, the hypermethylator phenotype of SDH-deficient GISTs^9^ was associated with the methylation of an insulator region (referred to as the “FGF insulator”) located in the upstream region of *FGF4* (between *FGF3* and *AP003555.2*). This epigenetic alteration caused genome topology changes, allowing *ANO1* (which has super-enhancer activity) to induce the expression of the *FGF4* oncogene^8^. *FGFR1* was found to be highly expressed in GISTs^7,8,10,11^, and activation of the downstream signalling of FGFR, through *AKT* or *MAPK*, was demonstrated both in quadruple WT and SDH-deficient GISTs, supporting the presence of an autocrine loop between *FGF4* and *FGFR1*^7,8^.

Together, these data highlight the involvement of *FGF4* in the biology of GISTs that do not rely on *KIT* or *PDGFRA*. However, how the *FGF4* duplication detected in quadruple WT could induce *FGF4* expression was not investigated. Thus, in the present work, we studied the methylation status and the structure of *FGF4* duplication in quadruple WT GISTs to assess whether epigenetic changes occurred and whether they could be involved in the regulation of *FGF4* expression.

## Material and methods

### Tumour samples

Tissue samples from GIST patients (fresh frozen or formalin-fixed paraffin-embedded already centralized at “Giorgio Prodi” Cancer Research Center (CIRC), University of Bologna) were studied. Diagnosis of GIST was based on histologic evaluation and immunohistochemistry of CD117 and DOG1. SDH deficiency was checked using IHC of SDHB. Quadruple WT cases were defined as being negative for mutations in the *KIT*, *PDGFRA*, *SDHA-B-C-D*, and *RAS*-P genes, which was confirmed by Sanger sequencing and next generation sequencing (NGS)^7^. Six quadruple WT cases positive for *FGF4* duplication were identified^7^ and used in the present study. Clinically, tumours were localized in the duodenum in four cases and in the ileum in two cases: two females and four males, with a median age of 60 years (range 44–73). From a molecular point of view, the presence of relevant gene fusions or somatic mutations was excluded through whole-exome and whole-transcriptome sequencing, with the exception of two patients in which mutations on tumour suppressor genes were identified: GIST127 carried a mutation in *CTNND2,* and GIST320 had *MEN1* and *TP53* mutations^5^.

As a cohort for comparison, we used GIST samples already present in our tissue bank and for which molecular data useful for this study were available: 26 SDH-deficient samples (15 mutated in *SDHA*, 4 in *SDHB* and 7 in *SDHC*) and 17 KIT mutant samples (10 mutated in exon 11 and 7 mutated in exon 9) and two quadruple WT GIST samples negative for *FGF4* duplication (described in^7^). The tumour samples analysed were all from primary tumours. This study was approved by the local institutional ethical committee of Azienda Ospedaliero-Universitaria Policlinico S. Orsola-Malpighi (number 113/2008/U/Tess). All experiments were performed in accordance with relevant guidelines/regulations, and informed consent was obtained from all participants.

### Global methylation profile

The global methylation profile was evaluated in 4 quadruple WT, 4 SDH-deficient (*3 SDHA mutant and 1 SDHC epimutant*) and 4 KIT mutant GISTs (exon 11 mutated). To correct for the different tumour localization of SDH-deficient and quadruple WT cases, KIT mutant cohort included both stomach and intestinal tumours. Genomic DNA was analysed using the Infinium Human Methylation 450 BeadChip (Illumina, San Diego, CA) following the manufacturer’s instructions. Data analysis was performed by adopting the approach described by Bacalini et al. (2015)^12^. Briefly, the Infinium 450k annotation was used to group the CpG probes on the same island/shelf/shore associated with the genes. For each gene, we evaluated the methylation changes by applying a multivariate analysis of variance (MANOVA) correcting for multiple hypothesis comparisons with Benjamini–Hochberg correction.

### Target methylation analysis

CpG methylation at specific loci was assessed through bisulfite conversion and enzymatic digestion on the six quadruple WT samples positive for *FGF4* duplication and on an expanded cohort of other molecular subgroups of GIST: 26 SDH-deficient cases, 17 KIT mutant cases, and two quadruple WT GISTs negative for FGF4 duplication. For bisulfite conversion, 200 ng of DNA was converted using the Methylamp DNA Modification Kit (Epigentek, Farmingdale, NY) following the manufacturer’s instructions. Then, 3 µl of converted DNA was amplified with primers specific for the FGF insulator region located at chr11:69,918,992–69,919,031 (kindly provided by Dr William Flavahan, Harvard Medical School, Boston, MA, USA). Amplicons were then sequenced using the BigDye Terminator v1.1 Cycle Sequencing Kit for Sanger sequencing (Thermo Fisher Scientific).

For enzymatic digestion, 100 ng of DNA was digested using the EpiJET DNA Methylation Analysis Kit (MspI/HpaII) (Thermo Fisher Scientific, Monza, Italy). In brief, DNA was digested with the MspI and HpaII restriction enzymes, two isoschizomers with differing sensitivities to CpG methylation. When CpG regions are methylated, cleavage with Epi HpaII is blocked, but cleavage with Epi MspI is not affected. HpaII is able to cleave only unmethylated DNA, while MspI cleaves both methylated and unmethylated DNA. qPCR using primers specific for the FGF insulator was performed on undigested (Cq1), HpaII digested (Cq2) or MspI digested (Cq3) DNA. The primers used were FW 5′-AATGTCCCCTGCACATGGAG-3′ and RV 5′-GCCGCGTCTCTCACATTTTC-3′. All samples were completely digested with the control enzyme MspI (threshold cycles Cq3 – Cq1 > 4.5) and were used for the analysis. The level of DNA methylation (%) was estimated using the formula: % of 5-mC = 100/(1 + E) Cq2 – Cq1, where E was the PCR efficiency value.

### Copy number

Copy number assessments were performed using TaqMan copy number assays (Thermo Fisher Scientific). Evaluation of the FGF insulator copy number was performed using Hs06280902_cn and Hs06332446_cn FAM‐labelled probes, covering the upstream and downstream regions of the insulator, respectively. The *FGF4* copy number was evaluated as previously described^7^. Hs03800758_cn and Hs03782780_cn FAM‐labelled probes targeting *ENDOD1* and *XXRA1* located outside *FGF4* duplication margins, respectively, were used for copy number normalization. TaqMan RNaseP Control Reagent (VIC‐labelled; Thermo Fisher Scientific) was used as an internal reference control. The copy number was estimated using the DDCt method and using a normal diploid sample as the calibrator. Moreover, high-density copy number array data (CytoScan HD or Oncoscan CNV Plus, Thermo Fisher Scientific) of quadruple WT GISTs were obtained from our previously published paper^7^.

### Sanger sequencing

Ten nanograms of DNA was used for amplification and sequencing of regions of interest: the FGF insulator locus and two SNPs located inside the UTR of *FGF4 (*rs3168175 in the 3′UTR and rs9666584 in the 5′UTR). For fusion gene validation, 500 ng of RNA was reverse-transcribed with SuperScript IV Reverse Transcriptase (Thermo Fisher Scientific) using random primers. Then, cDNA was used for amplification of fusion gene breakpoint regions or for the evaluation of *FGF4*-UTR SNP expression. The primers used are listed in Suppl. Table 1. Amplified regions were purified and sequenced using the BigDye Terminator 1.1 Cycle Sequencing Kit on the ABI 3730 Genetic Analyzer (Applied Biosystems).

### Gene expression

Raw RNA sequencing data were obtained from our previously published manuscripts^5,7^. Reads were aligned using the TopHat/BowTie pipeline. TPM normalization was used to evaluate gene expression levels. Fusion gene prediction was performed on RNA-seq data of six quadruple WT GISTs using Defuse, ChimeraScan and FusionMap software.

## Results

### Methylation analysis of quadruple WT GISTs

Overexpression of *FGF4* was reported in both quadruple WT and SDH-deficient GISTs^7,8^. Since it was shown that the hypermethylator phenotype of SDH-deficient GISTs was responsible for *FGF4* transcriptional activation^8^, we investigated for the first time the global DNA methylation profile of quadruple WT GISTs to uncover whether overexpression of *FGF4* could be regulated by the same epigenetic mechanism as SDH-deficient cases.

**Figure 1:**
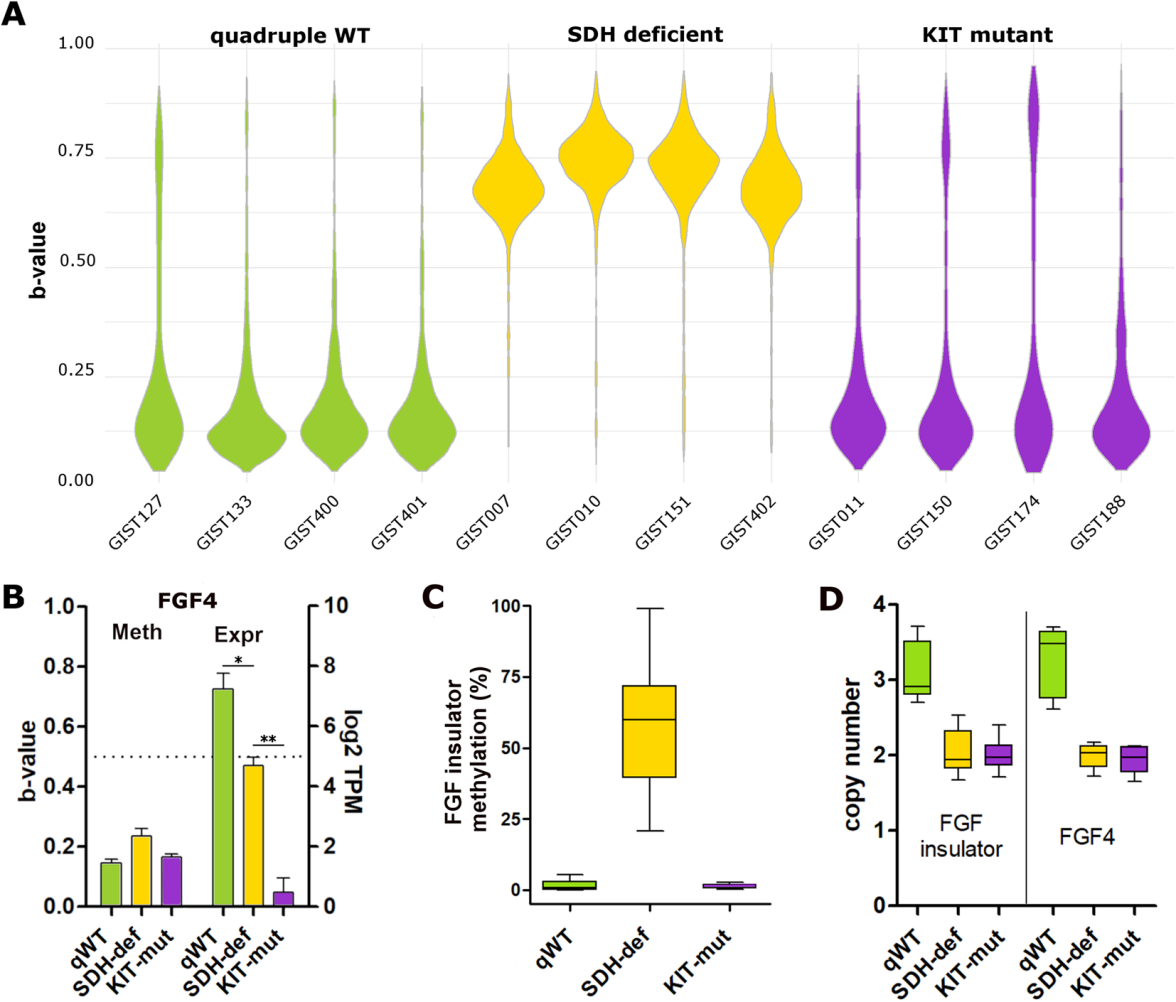
CpG methylation in quadruple WT and SDH-deficient GISTs. **(A)** Violin plot showing the B-value of global CpG methylation evaluated using the Infinium Human Methylation 450 BeadChip in GISTs. **(B)** Bar plot showing the absence of correlation between *FGF4* CpG methylation and *FGF4* mRNA expression. The B-value of the CpG island located at the *FGF4* locus is shown in the left panel, and the gene expression level (log2TPM) of *FGF4* is shown on the right. The t-test statistic was used (* < 0.05; *0.01). **(C)** Methylation level of the FGF insulator region evaluated through comparative enzymatic digestion in an extended cohort of GISTs (6 *FGF4*-positive quadruple WT, 26 SDH-deficient and 17 KIT mutant). **(D)** Box plot showing the copy number of the FGF insulator region (the two TaqMan assays, covering upstream and downstream regions of the insulator, were performed) of quadruple WT in comparison with KIT mutant and SDH-deficient GISTs. In quadruple WT cases, the FGF insulator was duplicated (three copies), which is similar to *FGF4*.

Global methylation analysis was performed on quadruple WT GISTs in comparison with SDH-deficient and KIT mutant cases. Quadruple WT cases showed a global methylation level similar to that of the KIT mutant, while SDH-deficient GISTs had significantly higher genomic methylation. This result indicated that the hypermethylator phenotype characteristic of SDH-deficient GISTs was not present in quadruple WT cases (Fig. 1A). Moreover, focusing on the *FGF4* locus, we showed that neither gene methylation was responsible for *FGF4* upregulation in quadruple WT GIST. In fact, CpG dinucleotides located inside the *FGF4* gene were hypomethylated in all GIST subgroups independent of *FGF4* gene expression, which was higher in SDH-deficient and quadruple WT GISTs and was absent in KIT mutant GISTs (Fig. 1B). This result indicated that in these tumours, the CpG methylation status of the *FGF4* locus was not responsible for its transcriptional activation. Moreover, as previously demonstrated^7^, *FGF4* expression seemed to be higher in quadruple WT cases than in SDH-deficient cases, suggesting that different activation mechanisms subtended *FGF4* induction in quadruple WT GIST.

Then, we explored the epigenetic status of the so-called FGF insulator described by Flavahan et al., whose hypermethylation in SDH-deficient GISTs causes the loss of functional chromatin boundaries, thus allowing the adjacent *ANO1* super-enhancer to contact and activate *FGF4*^8^. We confirmed in an extended cohort that the FGF insulator was methylated specifically in SDH-deficient with respect to other GIST subgroups (Fig. 1C), thus reinforcing the role of SDH loss in the alteration of the epigenetic status of *FGF4* in GIST. In contrast, it was fully unmethylated in both FGF4-positive quadruple WT and KIT mutant GISTs (Fig. 1C and Suppl. Figure 1), indicating that the methylation status of the FGF insulator was not the driver of the remarkably high expression of *FGF4* in these quadruple WT cases. Of note, the analysis was also performed on two additional quadruple WT GISTs negative for *FGF4* duplication and for *FGF4* expression (described in^7^), confirming the absence of methylation of the FGF insulator in this setting (data not shown). Since it was also demonstrated that disruption of this insulator by gene editing induces overexpression of *FGF4*^8^, we further assessed the integrity of the FGF insulator in quadruple WT cases in addition to methylation status. Through direct sequencing, we excluded the presence of mutations inside this region (not shown), and through copy number assay, we confirmed that the region was duplicated along with *FGF4* (Fig. 1D).

Altogether, these data reinforced the role of SDH loss in the alteration of the epigenetic status of *FGF4* in GISTs, while they excluded the possibility that *FGF4* overexpression in quadruple WT cases could be due to an epigenetic loss of the FGF insulator region. This result raised the question as to whether the genomic structure of the duplication could be responsible for *FGF4* transcriptional activation.

### Correlation between FGF4 duplication structure and FGF4 expression

To understand the molecular mechanism underlying the high transcriptional level of *FGF4* in quadruple WT GISTs, we first dissected the genomic structure and location of the duplicated *FGF4* allele.

**Figure 2:**
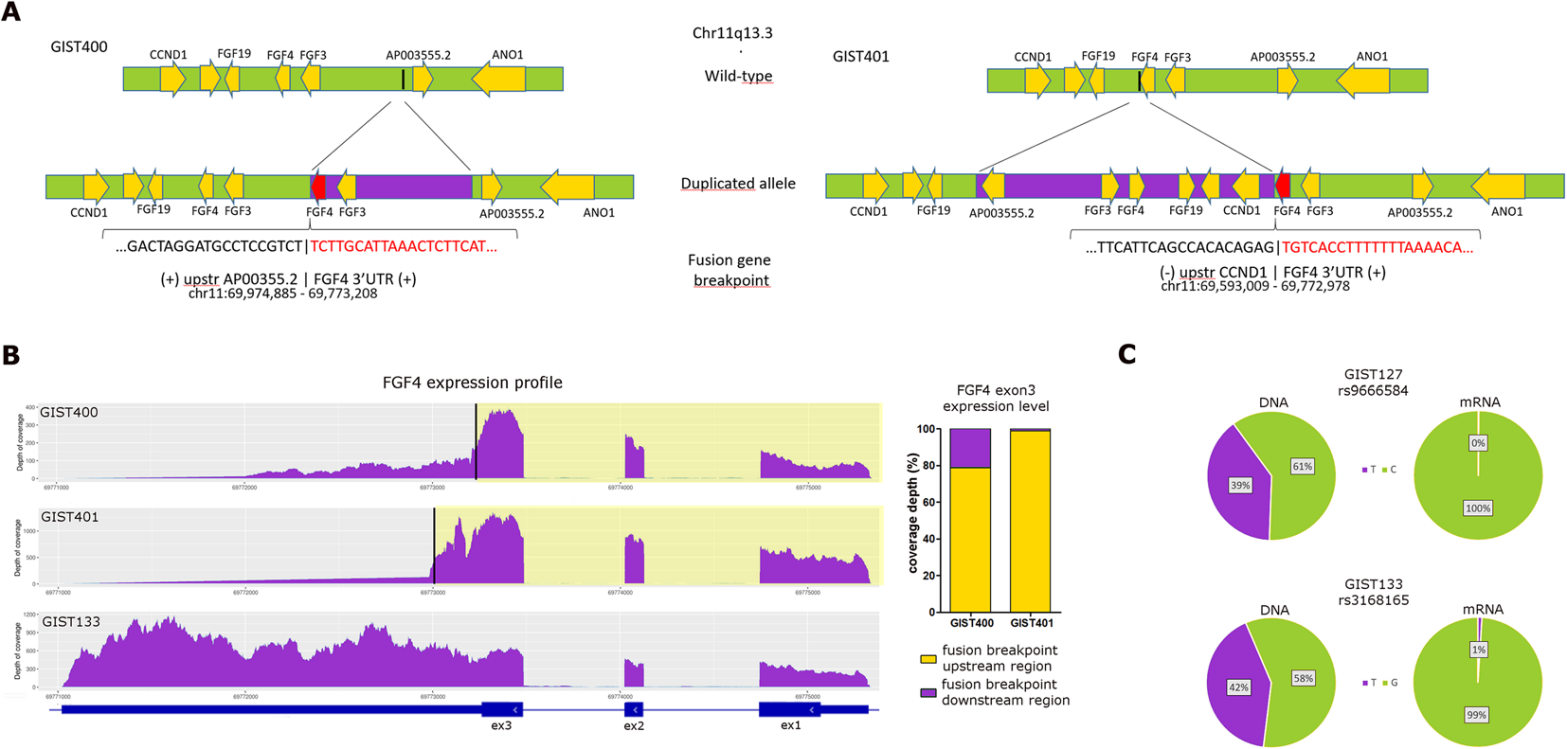
Structure of FGF4 duplication and monoallelic expression of *FGF4* mRNA. **(A)** Schematic representation of the structure of *FGF4* duplication in two quadruple WT cases. In GIST400 (left panel), the genomic region between *FGF4*-3UTR and the upstream region of *AP003555.2* was duplicated in tandem, producing the fusion gene *FGF4*-3UTR/*AP003555.2*-upstr. In GIST401 (right panel), the genomic region between *CCND1* and *AP003555.2* was duplicated, inverted and inserted into the 3′UTR of *FGF4*, producing the fusion gene *FGF4*-3UTR/*CCND1*-upstr. Duplicated regions are shown in violet. Genes are indicated by yellow arrows, depending on their transcriptional direction. The *FGF4* copy involved in the fusion is shown in red. Breakpoint sequence and position on the genome are shown for each fusion: sequences corresponding to *FGF4-3UTR* are shown in red (+ or − marks indicated if the sequence maps on the positive or negative strand). **(B)** Plot (in the left) showing the coverage per base of *FGF4* mRNA in the two quadruple WT cases carrying *FGF4*-3UTR fusion. The *FGF4* region retained in the fusion gene is highlighted in yellow. As a comparison, coverage of a quadruple WT without fusion (GIST133) is shown (lower panel). A relevant drop in coverage was detected in the cases carrying *FGF4*-3UTR fusion after the breakpoint of each case (indicated by a black bar). On the right, a box plot shows the coverage depth of exon 3 in *FGF4* before and after the fusion breakpoint. (**C**) Bar plot showing the allelic fraction (%) of two SNPs located at the *FGF4* locus (specifically rs3168175 in the 3′UTR and rs9666584 in the 5′UTR) at the DNA and mRNA levels in two quadruple WT GISTs (GIST127 and GIST133), indicating that FGF4 expression is monoallelic.

First, by exploiting previously published RNA-seq and copy number variation (CNV) information^7^, we investigated the genomic position of the duplicated copy of the *FGF4* gene. Fusion transcripts were predicted starting from RNA-seq data of the whole series of *FGF4*-positive quadruple WT GISTs, and RT-PCR was used for validation. Supplementary Table 2 shows all the predicted fusion transcripts involving chromosome 11. In two samples, GIST133 and GIST219, no fusions were detected. In the remaining samples, several fusions were identified, among which intrachromosomal rearrangements involving q13 cytobands of chromosome 11 were predominant, further indicating the high instability of the genomic region surrounding *FGF4* in quadruple WT GIST. GIST127 and GIST320 showed several consecutive structural alterations on chromosome 11 that were responsible for the majority of fusion genes identified in these samples (Suppl. Table 3). However, due to the complexity of the region and the absence of fusion genes directly affecting *FGF4*, it was not possible to properly dissect the structure of *FGF4* in these samples. Conversely, GIST400 and GIST401 harboured only one duplicated segment in chr11, the one encompassing *FGF4*. Moreover, two fusion genes involving the terminal untranslated region (3′UTR) of *FGF4* were detected in these two tumours: *FGF4-3UTR* was fused with the intergenic region upstream of *CCND1* in GIST401 and with the upstream region of *AP003555.2* in GIST400. Breakpoints of these fusions corresponded approximatively to the boundaries of the duplication events detected (Suppl. Table 3, Fig. 2A), indicating that these fusion genes were produced by the insertion of the duplicated copy of *FGF4*. Thus, it was possible to demonstrate that the duplicated allele was integrated in the same chromosome and in close proximity to the parental copy of *FGF4*. Therefore, in these two GISTs, by combining information from RNA sequencing and CNV analysis, we can describe the structure of the duplication, in which *FGF4* is either inserted in tandem in the intergenic region between *FGF3* and *AP003555.2* (GIST_400) or is fused with the inverted upstream region of *CCND1* (GIST_401) (Fig. 2A).

Finally, we investigated whether *FGF4* expression was uniformly upregulated or whether its overexpression was due to a single rearranged allele. Interestingly, we discovered that only one of the three copies of *FGF4* was predominantly expressed. In the two quadruple WT GISTs harbouring *FGF4*-3UTR fusion genes, we observed that the depth of RNA-seq reads over *FGF4* had a relevant drop immediately after the breakpoint region of each case, indicating that the copy involved in the fusion was preferentially transcriptionally activated (Fig. 2B). Interestingly, in both cases, the copy of *FGF4* expressed and involved in the fusion was the one closer to *ANO1*. Similarly, among the other quadruple WT samples, it was possible to identify two informative heterozygous SNPs at the DNA level located in UTR regions of FGF4 that were monoallelically expressed in two cases (GIST127 and GIST133) (Fig. 2C), indicating that *FGF4* expression was derived from only one copy of *FGF4* also in these tumours. Globally, these findings indicated that *FGF4* overexpression detected in quadruple WT is a distinctive and recurrent feature that was not due to an epigenetic mechanism but rather to the specific genomic structure of the 11q13 duplication that induces the transcriptional activation of one allele of *FGF4*.

## Discussion

*FGFR1* is one of the most highly expressed tyrosine kinase receptors in GISTs after *KIT* and *PDGFRA*^7,8,10,11^. It has been reported that quadruple WT GISTs, which do not rely on *KIT* or *PDGFRA*, may present *FGFR1* activating mutations and fusion events^5,6^. Recently, in two different papers, overexpression of an FGFR1 ligand, FGF4, has been reported as a novel molecular event in two subgroups of GISTs without *KIT* and *PDGFRA* mutations, both SDH-deficient and quadruple WT GISTs. Moreover, activation of FGFR downstream signalling pathways through AKT and MAPK was demonstrated^7,8^.

In particular, Flavahan et al. reported that in SDH-deficient GISTs, the hypermethylator phenotype induced by SDH loss^9^ disrupts the binding of CTCF in regions located in proximity to the *FGF3*/*FGF4* locus, causing *FGF4* overexpression^8^. Generally, CTCF binding sites demarcate the boundaries of topologically associated domains (TADs), which are contiguous genomic intervals in which the majority of loci interact more frequently with each other than with loci outside the TAD. Thus, the disruption of these CTCF binding sites inactivates their insulator function, allowing contact with the adjacent TAD^13^. The CTCF binding site identified by Flavahan et al. was demonstrated to have an insulator function on *FGF3* and *FGF4,* and its hypermethylation allowed contact between the adjacent *ANO1* super-enhancer and FGF genes, causing the overexpression of *FGF4*^8^.

Conversely, we had previously reported that *FGF4* was recurrently overexpressed in quadruple WT GISTs in association with a focal duplication encompassing the gene. Duplication and overexpression of *FGF4* were mutually exclusive with other relevant alterations, and they were not detected in GISTs harbouring *FGFR1* mutations, supporting the pathogenic role of the event^7^. In our case series of quadruple WT GISTs, we investigated the epigenetic mechanisms modulating *FGF4* expression identified in SDH-deficient GISTs^8^, and we demonstrated that aberrant methylation was not responsible for *FGF4* overexpression in quadruple WT cases. Moreover, we found that *FGF4* expression was derived from the duplicated allele, preferentially from the copy nearest to *ANO1*. This suggests that the mechanism of upregulation of *FGF4* in quadruple WT GISTs is related to the genomic structure alteration caused by the duplication. One hypothesis is that the insertion of the duplicated allele in the proximity of *FGF4* could alter the chromatin conformation, allowing *FGF4* transcription to be induced by the super-enhancer activity of *ANO1*^8^. It has been shown that TAD boundaries are altered in a cancer-specific manner for each tumour type, presumably reflecting the distribution of cancer driver genes^14^. In cancer, duplication events frequently extend over the neighbouring TAD (inter-TAD), resulting in the formation of new chromatin domains (neo-TAD) that could alter the expression level of the involved genes^14–16^. Since the duplicated region identified in quadruple WT GISTs is involved at least two neighbouring TADs^8^, it is possible that the resulting neo-TAD formation allowed for the interaction with the *ANO1* super-enhancer inducing the aberrant expression of *FGF4*. Further studies are needed to confirm this hypothesis; however, the extreme rarity of these tumours, the extensive molecular characterization already performed and the lack of appropriate cellular models hamper the development of deeper analyses on this aspect.

Even if the mechanism underlying *FGF4* upregulation seems to be different between SDH-deficient and quadruple WT GISTs, a common altered signalling pathway could be identified, and the therapeutic consequences for patients could be similar. Taken together, our findings indicated that FGFR pathway alteration is a common oncogenic event in these two groups of *KIT/PDGFRA* WT GISTs, and targeted therapeutic approaches should be considered^10^. In vitro models of *KIT/PDGFRA* WT GISTs are still lacking, but Flavahan et al. developed an SDH-deficient GIST PDX model carrying epigenetic alterations and consequent overexpression of *FGF4* and demonstrated that it was responsive to BGJ-398, a selective inhibitor of FGFR1–4. This study further strengthened the biological relevance of FGFR signalling in the tumorigenesis of GISTs not dependent on *KIT* and *PDGFRA*, indicating a potential therapeutic option for these tumours^8^. Currently, no specific FGFR inhibitors are approved for GISTs. Regorafenib, which was approved in GISTs resistant to imatinib and sunitinib, is the only available drug that may also have a weak potential inhibition of FGFR. In fact, in vitro studies demonstrated that regorafenib also inhibited FGFR signalling in MCF‐7 breast cancer by inhibiting phosphorylated FGFR substrate 2 (pFRS2) and the downstream signalling kinase pERK1/2^17^. SDH-deficient GISTs were shown to obtain durable benefits from regofarenib treatment, but this positive result probably depends more on the inhibition of the KIT pathway and angiogenic kinases (VEGFR1/3)^18^. The ongoing clinical trial REGISTRI (NCT02638766) is evaluating whether WT KIT/PDGFRA may benefit from regorafenib upfront in first-line therapy; however, at present, no definitive conclusion can be drawn due to the slow accrual. In addition to regorafenib, in the last decade, other multi-TKIs active against FGFR (e.g., dovitinib, masitinib, ponatinib and pazopanib) have also been tested in GISTs, and some of them have produced encouraging results^10,19,20^. However, all these trials were predominantly conducted on mutant KIT GISTs, and no conclusion can be drawn about the efficacy of these multi-TKIs in rarer mutational subsets of GISTs.

In conclusion, we demonstrated the presence of a novel mechanism of *FGF4* regulation in quadruple WT GISTs. Even if the genetic alteration is different, overexpression of the *FGF4* oncogene is a common event in both SDH-deficient and quadruple WT GISTs, indicating for the first time a putative common therapeutic target for GISTs that do not rely on KIT/PDGFRA/RAS-P activation. Clinical experience or trials specific for KIT/PDGFRA/RAS-P WT GISTs using FGFR inhibitors should be encouraged.

## Supplementary information

Supplementary Information.

## Data Availability

The datasets generated during and/or analysed during the current study are available from the corresponding author on reasonable request.

## Abbreviations

CNV: Copy number variation
GIST: Gastrointestinal stromal tumours
TAD: Topologically associated domains
WT: Wild-type
