# Supplementary material for: Gene duplication, rather than epigenetic changes, drives FGF4 overexpression in KIT/PDGFRA/SDH/RAS-P WT GIST
Source: Sci Rep. 2020 Nov 16;10:19829. doi: 10.1038/s41598-020-76519-y (PMC7670422; doi:10.1038/s41598-020-76519-y)
Supplement: Supplementary file 1 — Supplementary Information. [file 41598_2020_76519_MOESM1_ESM.pdf]

Title:

Gene duplication, rather than epigenetic changes, drives FGF4 overexpression in KIT/PDGFRA/SDH/RAS-P WT GIST

Authors:

Milena Urbini[1], Annalisa Astolfi[2*], Valentina Indio[3], Margherita Nannini[4], Angela Schipani[3,5], Maria Giulia Bacalini[6], Sabrina Angelini[7], Gloria Ravegnini[7], Giovanni Calice[8], Massimo Del Gaudio[9], Paola Secchiero[2], Paola Ulivi[1], Elisa Gruppioni[10], Maria Abbondanza Pantaleo [4,5,3]

Affiliations:

[1] Biosciences Laboratory, Istituto Scientifico Romagnolo per lo Studio e la Cura dei Tumori (IRST) IRCCS, Meldola, Italy

[2] Department of Morphology, Surgery and Experimental Medicine, University of Ferrara, Ferrara, Italy

[3] "Giorgio Prodi" Cancer Research Center (CIRC), University of Bologna, Bologna, Italy

[4] Division of Oncology, Azienda Ospedaliero Universitaria di Bologna, Via Albertoni 15, Bologna, Italy

[5] Department of Experimental, Diagnostic and Specialized Medicine, University of Bologna, Italy

[6] IRCCS Istituto delle Scienze Neurologiche di Bologna, 40139 Bologna, Italy.

[7] Department of Pharmacy and Biotechnology, University of Bologna, Bologna, Italy

[8] Laboratory of Preclinical and Translational Research, IRCCS-Referral Cancer Center of Basilicata (CROB), 85028 Rionero in Vulture (PZ), Italy.

[9] Department of Organ Insufficiencies and Transplantation, General Surgery and Transplantation, S. Orsola-Malpighi University Hospital, 40138 Bologna, Italy.

[10] Laboratory of Oncologic Molecular Pathology, S. Orsola-Malpighi Hospital, Bologna, Italy

Corresponding
Annalisa Astolfi: Department of Morphology, Surgery and Experimental Medicine, University of Ferrara, Ferrara, Italy; annalisa.astolfi@unife.it

Suppl. Fig 1

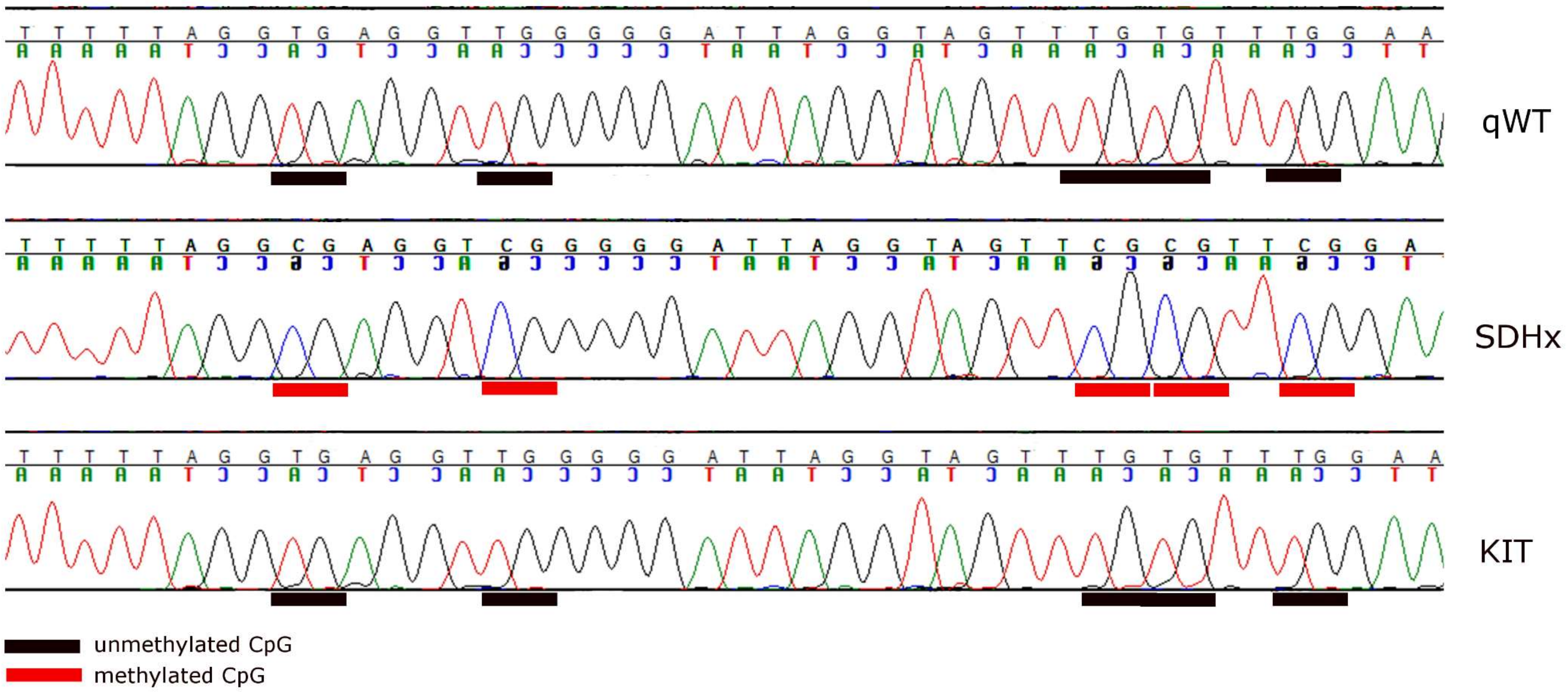

Supplementary Figure 1. Bisulfite sequencing of FGF insulator region. DNA was converted and FGF insulator peak (chr11:69,918,992-69,919,031) was sequenced. CG dinucleotide were indicated with bars: black for unmethylated (bisulfite converted to TG) and red for methylated (not converted) qWT=quadruple WT; SDHx = SDH-deficient GIST; KIT = KIT-mutant GIST

Supplementary Table 1. Primers used

Target	sequence 5'-3'
FGF insulator	
FGF insulator Fw1	CATTAGCCAAGAAAGAGAGGAGGG
FGF insulator Rev1	GGGAACAGTCTATGTCTCAGAGCTG
FGF insulator Fw2	CCGACCTGGTACACAGTGGGTG
FGF insulator Rev2	GCCTGTGAGTGAAGTGAAGGAGC
Fusion genes	
FGF4_3UTR_Fw	GGTGCACTTTCTTCGGATGAA
CCND1_Upstr_Rev	GAGCAGACGTGGCCTCCTCT
AP003555.2_Upstr_Rev	AGAAGGAAGATGCCAAGACCAG
rs9666584	
FGF4_5UTR_Fw	TCCATGCAGCCGGGGTAGAG
FGF4_5UTR_Rv	CAGCAAGGCCAGCAGGAC
rs3168175	
FGF4_3UTR_Fw2	CCATGATTGTCTTAAATGCCTTAAA
FGF4_3UTR_Rv	CGGTCAGCATGTCAATGTG

Suppl.Table 2. List of predicted fusion genes involving chromosome 11

					breakpoint position gene 1			breakpoint position gene 2				
sample		gene1	gene2	type	genomic	cytoband	gene_location	genomic	cytoband	gene_location	splitr_count	span_count
GIST400		FGF4	AP003555.2	intrachromosomal	chr11:69.773.208	q13.3	utr3p	chr11:69.974.885	q13.3	upstream	53	27
GIST401		CCND1	FGF4	intrachromosomal	chr11:69.593.009	q13.3	upstream	chr11:69.772.978	q13.3	utr3p	906	434
GIST127		PLA2G16	ATL3	intrachromosomal	chr11:63.614.000	q13.1	coding	chr11:63.659.252	q13.1	coding	18	16
		PPFIA1	MARK2	intrachromosomal	chr11:70.324.402	q13.3	coding	chr11:63.839.560	q13.1	intron	23	5
GIST320	*	SHANK2	PLAAT5	intrachromosomal	chr11:70.896.501	q13.4	coding	chr11:63.463.595	q12.3	coding	6	2
		FGF3	SHANK2	intrachromosomal	chr11:69.816.320	q13.3	coding	chr11:70.698.763	q13.4	coding	120	12
	*	LRP5	SHANK2	intrachromosomal	chr11:68.365.702	q13.2	coding	chr11:71.147.338	q13.4	coding	3	2
		SHANK2	USP43	interchromosomal	chr11:70.659.828	q13.4	coding	chr17:9.656.403	p13.1	coding	59	8
	*	ANO1	SLC22A9	intrachromosomal	chr11:70.171.038	q13.3	coding	chr11:63.370.495	q12.3	intron	29	5

* RT-PCR validation not performed

Supplementary Table 3. Structural alterations indentified in chr11q of 6 quadruple WT GIST. All events are gains of one copy of DNA and margins of marked regions (#) approximately corresponded to fusion genes breakpoints. In addition, loss of the entire q-arm of chromosome 11 was detected in GIST127 and GIST320

File	CN state	Chr	Cytoband Start	Cytoband End	Size (kbp)	Gene Count	Genes	Full Location (hg38)	
GIST127	Gain	11	q13.2	q13.3	1507664	24	NDUFS8, MIR7113, MIR4691, TCIRG1, MIR6753, CHKA, KMT5B, C11orf24, LRP5, PPP6R3, GAL, TESMIN, CPT1A, MRPL21, IGHMBP2, MRGPRD, MRGPRF, MRGPRF-AS1, TPCN2, MIR3164, LOC338694, MYEOV, LOC102724265, LINC01488	chr11:68031676-69539340	
GIST127	Gain	11	q13.3	q13.3	587919	8	ORAOV1, FGF19, FGF4, FGF3, LOC101928443, ANO1-AS2, ANO1, FADD	chr11:69660833 -70248752	
GIST127	Gain	11	q13.3	q13.4	2592443	44	PPFIA1, CTTN, SHANK2, SHANK2-AS1, SHANK2-AS3, MIR3664, FLJ42102, DHCR7, NADSYN1, MIR6754, KRTAP5-7, KRTAP5-8, KRTAP5-9, KRTAP5-10, KRTAP5-11, FAM86C1, ALG1L9P, ZNF705E, DEFB108B, LOC100133315, LOC100129216, RNF121, IL18BP, NUMA1, LOC100128494, MIR3165, LRTOMT, LAMTOR1, ANAPC15, FOLR3, FOLR1, FOLR2, INPPL1, PHOX2A, CLPB, LINC01537, PDE2A, MIR139, ARAP1, STARD10, MIR4692, ATG16L2, FCHSD2, MIR4459	chr11:70292238-72884681	#
GIST133	Gain	11	q13.3	q13.3	213984	2	FGF4, FGF3	chr11:69771253 -69953898	
GIST 219	Gain	11	q13.3	q13.3	149222	2	FGF4, FGF3	chr11:69749999 -69899221	
GIST 320	Gain	11	q13.2	q13.2	1035900	35	RAD9A, PPP1CA, TBC1D10C, CARNS1, RPS6KB2, PTPRCAP, CORO1B, GPR152, CABP4, TMEM134, AIP, MIR6752, PITPNM1, CDK2AP2, CABP2, GSTP1, C11orf72, NDUFV1, DOC2GP, NUDT8, TBX10, ACY3, ALDH3B2, FAM86C2P, UNC93B1, ALDH3B1, NDUFS8, MIR7113, MIR4691, TCIRG1, MIR6753, CHKA, KMT5B, C11orf24, LRP5	chr11:67393462 -68429362	#
GIST 320	Gain	11	q13.3	q13.3	62154	2	CCND1, ORAOV1	chr11:69628723 -69690877	
GIST 320	Gain	11	q13.3	q13.3	434979	6	FGF4, FGF3, LOC101928443, ANO1-AS2, ANO1, FADD	chr11:69772966 -70207945	#
GIST 320	Gain	11	q13.4	q13.4	92755	2	SHANK2, SHANK2-AS1	chr11:70630705 -70723460	#
GIST400	Gain	11	q13.3	q13.3	217406	2	FGF4, FGF3	chr11:69771253 -69957320	#
GIST401	Gain	11	q13.3	q13.3	458308	5	CCND1, ORAOV1, FGF19, FGF4, FGF3	chr11:69600942 -70027911	#
